# The gut–meningeal immune axis: Priming brain defense against the most likely invaders

**DOI:** 10.1084/jem.20211520

**Published:** 2022-02-23

**Authors:** Rafael Di Marco Barros, Zachary Fitzpatrick, Menna R. Clatworthy

**Affiliations:** 1 Molecular Immunity Unit, Department of Medicine, University of Cambridge, Cambridge, UK; 2 Spark Therapeutics, Philadelphia, PA; 3 Cambridge Institute of Therapeutic Immunology and Infectious Diseases, University of Cambridge, Cambridge, UK; 4 Cellular Genetics, Wellcome Sanger Institute, Hinxton, UK

## Abstract

The gastrointestinal tract contains trillions of microorganisms that exist symbiotically with the host due to a tolerant, regulatory cell–rich intestinal immune system. However, this intimate relationship with the microbiome inevitably comes with risks, with intestinal organisms being the most common cause of bacteremia. The vasculature of the brain-lining meninges contains fenestrated endothelium, conferring vulnerability to invasion by circulating microbes. We propose that this has evolutionarily led to close links between gut and meningeal immunity, to prime the central nervous system defense against the most likely invaders. This paradigm is exemplified by the dural venous sinus IgA defense system, where the antibody repertoire mirrors that of the gut.

## Teaser

The vasculature of the meninges contains fenestrated endothelium conferring vulnerability to invasion by circulating microbes, often arising from the gut. Here, we discuss how close links between intestinal and meningeal immunity enable central nervous system defense against blood-borne invaders.

## Introduction

The central nervous system (CNS), comprising the brain and spinal cord, is arguably the most important of all organ systems, housing the centers that control respiration and heart rate, movement and sensation, higher thought, and reason. In recent years, there has been an explosion of interest in the immunology of CNS-adjacent tissues such as the meninges, the membranes that cover the outer surface of the CNS (Mastorakos and McGavern, 2019). A number of studies have described the presence of innate and adaptive immune cells in the meninges (Mrdjen et al., 2018; Van Hove et al., 2019), and the importance of defining their number, nature, and function lies in their proximity to the brain and their potential to influence brain cell activity and health in homeostasis and disease (Alves de Lima et al., 2020). Meningeal immune cells respond to CNS-intrinsic perturbations, such as neuroinflammation or neurodegeneration (Da Mesquita et al., 2018; Da Mesquita et al., 2021; Louveau et al., 2018), as well as to CNS-extrinsic challenges, such as systemic infection (Fitzpatrick et al., 2020). The meninges may also provide a site for negative selection of CNS antigen-specific developing B cells (Brioschi et al., 2021; Schafflick et al., 2021; Wang et al., 2021). While the majority of studies profiling the function of meningeal immune cells have focused on responses to CNS pathologies, evolutionarily, immune challenges that come from “without” rather than “within” are more likely to be the selective pressure that has shaped where and how meningeal immune cells act; put another way, diseases like multiple sclerosis or Alzheimer’s disease affect humans at an age when reproduction is completed, whereas viral/bacterial meningitis and encephalitis kill infants and children (GBD 2016 Meningitis Collaborators, 2018), and therefore likely represent a powerful evolutionary force that has shaped meningeal immunity to optimize defense against such pathogens. We propose that this concept lies at the heart of why peripheral organs, particularly the gastrointestinal tract, can, and do, shape meningeal immunity.

## The meninges: The peripheral interface of the CNS

**Figure 1.**
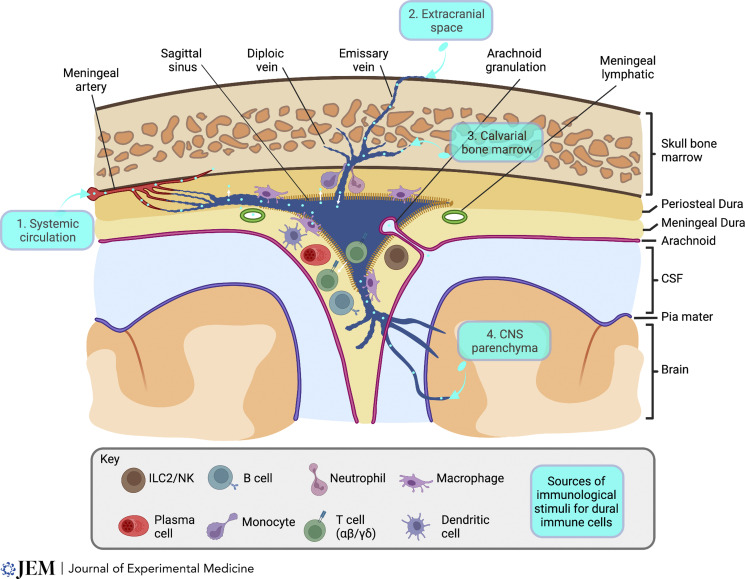
**The meninges: The peripheral interface of the CNS.** The meninges are composed of the dura, arachnoid, and pia mater. The dura has a bone-lining periosteal layer and an inner serous/meningeal layer that separate to accommodate the dural venous sinuses. Dural immune cells are concentrated around the wall of the dural venous sinuses, and macrophages are closely opposed to blood vessels. The arterial blood supply of the dura comes from the middle meningeal artery, with branches supplying arterial blood to the overlying calvarium and underlying dura, feeding capillary beds that drain into dural veins and ultimately into the dural venous sinus. Diploic veins drain the bone/bone marrow, and emissary veins drain the extra-cranial space into the venous sinuses. Unlike the vascular network of the CNS, which is sealed by endothelial cell tight junctions, the dural vascular bed is fenestrated, enabling blood-borne molecules and immune cells to exit the vasculature. This provides a direct means by which physically distant organs, such as the gut, may communicate with meningeal immune cells, or indeed, a potential route of entry by which systemic immune cells (including those stimulated or educated at a distal site) may enter the dura. The dural immune sinuses also receive immunological information from the CNS as CSF may drain into the sinuses via arachnoid granulations.

The meninges of higher vertebrates are composed of an outer dura mater, a middle arachnoid mater, and an inner pia mater. They lie at the interface between the brain and its surrounding bony box, including the calvarium, the bones that form the internal wall of facial structures, like the air sinuses, and those at the base of the skull. The dura mater, the toughest and thickest of the three layers, lies immediately adjacent to the internal surface of the skull bones and vertebral spinal canal and has large folds that dip between the right and left cerebral and cerebellar hemispheres (falx cerebri and falx/tentorium cerebelli, respectively). The dura has a bone-lining periosteal layer and an inner meningeal/serous layer that separate to accommodate the dural venous sinuses (Fig. 1) around which immune cells congregate (Fitzpatrick et al., 2020; Rustenhoven et al., 2021). It is notable that blood flows relatively slowly through the dural sinuses and the endothelium is fenestrated (Kilic and Akakin, 2008; Schuchardt et al., 2015), providing ample opportunity for the delivery of immunological information to adjacent immune cells. Immunological cues may derive from the local tissues drained by the dural venous sinuses (namely, the skull and vertebral bones, the meninges, and the brain and spinal cord), alerting immune cells to CNS “intrinsic” perturbations, but may also include systemically generated immune stimuli, cells, or blood-borne microbes. In fact, lymphocytes have been directly visualized exiting the superior sagittal sinus into the parasagittal space in vivo (Rustenhoven et al., 2021). The dura mater has particularly close links with the calvarial bone marrow via bony channels through which diploic veins (draining the bone) and emissary veins (draining the extracranial space) transit en route to the venous sinuses (Fig. 1; Mortazavi et al., 2012; Tsutsumi et al., 2013). Indeed, cells from the bone marrow, including neutrophils and monocytes, may directly transit to the meninges and brain parenchyma through vascular channels following a stroke or aseptic meningitis (Cai et al., 2019; Cugurra et al., 2021; Herisson et al., 2018; Yao et al., 2018). More recent data suggest that developing B cells may also traffic to the dura from the skull bone marrow (Brioschi et al., 2021; Schafflick et al., 2021; Wang et al., 2021). As well as these channels that accommodate diploic and emissary veins, the bones at the base of the skull have holes (foramena) through which cranial nerves pass, potentially exposing the meninges to extracranial challenges.

The dura itself mostly derives its arterial blood supply from the middle meningeal artery (a branch of the external carotid artery), which feeds a vascular network that runs between the periosteal dura and skull bone. Some of these branches supply arterial blood to the overlying bone, whereas others give rise to precapillary arterioles that penetrate the dura mater feeding into capillary beds (Roland et al., 1987). Dural veins are similarly arranged into a deep and superficial network, with superficial veins running alongside the meningeal arteries and deep veins forming an irregular network within the dura mater before draining into venous sinuses (Fig. 1). Unlike the vascular network of the CNS, which is sealed by endothelial cell tight junctions, the dural vascular bed is fenestrated (Derk et al., 2021). Consistent with this, intravenous horseradish peroxidase (44 kD) accumulated throughout the dura, but not the cerebral parenchyma, in rodents and monkeys (Balin et al., 1986). Furthermore, endothelial cells from mouse dura mater showed limited expression of *Claudin-5* and *Occludin*, two tight junction components that are highly expressed in mouse brain vascular endothelial cells (Dejana, 2004; Rustenhoven et al., 2021). Endothelial fenestrations have been directly visualized in dural veins using electron microscopy (Nabeshima et al., 1975). Thus, the fenestrations of the dural vasculature expose the dura to blood-borne microbes and provide a direct means by which physically distant organs, such as the gut, may communicate with meningeal immune cells, or indeed, a potential route of entry by which systemic immune cells (including those stimulated or educated at a distal site) may enter the dura.

The arachnoid mater is a thin avascular membrane made up of squamous epithelial cells bound by tight junctions and desmosomes, while the inner pia mater adheres to the surface of the brain (Fig. 1) and forms a selectively permeable barrier between the cerebral parenchyma and the sub-arachnoid space (Alcolado et al., 1988; Hannocks et al., 2018). The sub-arachnoid space contains cerebrospinal fluid (CSF), which constitutively undergoes macromolecular exchange with the interstitial fluid of CNS parenchyma via the paravascular spaces (“glymphatics”) of CNS-penetrating vessels (Iliff et al., 2012; Lun et al., 2015). Antigenic sampling of CSF represents a potential mechanism of CNS immunosurveillance, but there is considerable debate as to the anatomical route(s) through which CSF is drained (see recent review [Proulx, 2021]). Historically, the prevailing view has been that CSF drains directly into the dural venous sinus via the arachnoid granulations (Proulx, 2021), bringing CNS-derived antigens or immune mediators in close proximity with sinus-adjacent immune cells. However, tracer dyes, contrast agents, proteins, and even immune cells injected into sub-arachnoid space, cerebral ventricles, and cerebral parenchyma readily accumulate in deep cervical LNs (dCLNs; Ahn et al., 2019; Antila et al., 2017; Aspelund et al., 2015; Da Mesquita et al., 2018; Louveau et al., 2018; Louveau et al., 2015; Ma et al., 2017). Drainage of CSF into the dCLN may take place via meningeal lymphatic vessels (Aspelund et al., 2015; Da Mesquita et al., 2018; Louveau et al., 2018; Louveau et al., 2015) or via extracranial lymphatic vessels (Ahn et al., 2019; Ma et al., 2017; Proulx, 2021). In addition, CSF tracers can accumulate in the parasagittal dura independently of meningeal lymphatic vessels (Ringstad and Eide, 2020; Rustenhoven et al., 2021). Therefore, there are likely multiple pathways for CSF drainage, and it is currently unclear how immune cells in the dCLNs differentiate between antigens derived from CSF versus other head and neck sites.

## Meningeal immune cells occupy areas of need

Meningeal immune cells in homeostasis are most numerous in the dura mater and have been predominantly profiled by generating single-cell suspensions of mouse dura. Flow and mass cytometry studies, as well as single-cell RNA sequencing, have identified a variety of immune cells, including neutrophils, mast cells, monocytes, macrophages, dendritic cells, innate lymphocytes (particularly natural killer [NK] cells, and groups 2 and 3 innate lymphoid cells [ILCs]), γδ T cells, NK T cells, CD4 and CD8 T cells, B cells, and plasma cells (Korin et al., 2017; Mrdjen et al., 2018; Van Hove et al., 2019). However, some methodological limitations should be borne in mind when interpreting these data: First, it is difficult to exclude contamination of cells from the skull bone marrow, as there are several channels that connect the bone marrow and dura. Second, unless an intravenous anti-CD45 antibody is administered prior to dural harvest, the single-cell suspension obtained will inevitably contain intravascular contaminants as well as dural-resident immune cells, even if animals are perfused. Third, some cells are not well represented in single-cell suspensions as they are more closely intertwined within the structural components of the dura and are not released by standard enzymatic/mechanical digestions.

Imaging-based studies overcome some of these limitations and enable the precise location of cells to be described. Such studies have revealed that the vast majority of the area occupied by the dura is pauci-immune, but immune cells are enriched in specific regions, particularly the walls of the dural venous sinuses (Fig. 1), with ILC2 (Gadani et al., 2017), γδT cells (Ribeiro et al., 2019), B cells, and plasma cells (Fitzpatrick et al., 2020), as well as CD8 and CD4 T cells, NK T cells, and Foxp3+ regulatory T cells (Rustenhoven et al., 2021) noted in the peri-sinus area of the superior sagittal and transverse sinuses. Within the dura, the vascular network is guarded by macrophages (Kierdorf et al., 2019) that may be replaced by monocyte-derived macrophages during intra-cranial viral infection (Rua et al., 2019). These perivascular macrophages cluster along the dural sinuses and the veins that feed into them (Rua et al., 2019; Sato et al., 2021), alongside arterioles (Rustenhoven et al., 2021), and straddle dural nerves (Sato et al., 2021). Dural macrophages are largely non-motile, constitutively survey their surrounding microenvironment by extending and retracting dendrites (Kierdorf et al., 2019), but mobilize to encircle damaged vasculature following traumatic brain injury (Mastorakos et al., 2021). Peri-sinus macrophages and dendritic cells constitutively sample antigens from the circulation and CSF, evidenced by their uptake of fluorescently tagged OVA injected intravenously and/or into the cisterna magna (Rustenhoven et al., 2021). Overall, the anatomical position of immune cells in the meninges around blood vessels and dural venous sinuses are tailored to bolster defense in vulnerable barrier areas that interface with the periphery via fenestrated endothelium.

## Where do CNS invaders arise?

If neurotropic and leptomeningeal infections have driven the evolution of meningeal immunosurveillance, it is informative to consider the most prevalent CNS pathogens and their site of origin. Such infections account for up to ∼4% of global deaths in pediatric populations under 9 years of age (GBD 2016 Meningitis Collaborators, 2018). These infections arise either from local invasion or hematogenous spread. In terms of the former, the meninges provide an important barrier to the extracranial environment, to which the CNS is exposed across the various skull base foramina that accommodate the exit of cranial nerves. This defense system is largely effective, but disruption of meningeal integrity by traumatic skill base fractures (evident by the appearance of a CSF leak) is associated with an elevated risk of acute bacterial meningitis (Ter Horst et al., 2020) and of long-term recurrent bacterial meningitis (Adriani et al., 2007). Similarly, a rare complication of dural puncture is iatrogenic meningitis caused by Viridans-type Streptococci transmitted during instrumentation (Baer, 2006). Meningitis can also develop as a consequence of direct bacterial invasion in the context of severe soft tissue/bone infection in adjacent anatomical structures. Hence, mastoiditis (Barry et al., 2019), orbital cellulitis, and sinusitis (Ziegler et al., 2018) can each be complicated by meningitis. Thus, the meningeal barrier is critical for host CNS defense against local invasion, and when normal anatomy is intact, it is largely effective in achieving this, but disruption in the context of traumatic injury or procedural/surgical interventions can be associated with life-threatening infection.

In addition to defending against local invaders, the meninges must also protect the CNS from infectious challenges that metastasize via the hematogenous route. Such hematogenous spread is thought to account for the majority of community-acquired bacterial meningitis (van de Beek et al., 2021), and given that dural vascular endothelium is fenestrated, it is an area of vulnerability for the CNS, as discussed previously. The underlying microbial causes of meningitis vary with patient demographics, clinical context, and environmental exposure. However, it is notable that the majority of these blood-borne infections are thought to derive from the respiratory tract (van de Beek et al., 2021). Hence, Pneumococcus, Meningococcus, and *Haemophilus influenzae B* are globally the most common causes of bacterial community-acquired meningitis, and enter the host bloodstream via the respiratory tract mucosa (van de Beek et al., 2021). Gut bacteria do not commonly cause meningitis (Unhanand et al., 1993; van de Beek et al., 2021). Exceptions include *Listeria monocytogenes*, which can cause severe CNS infection in the immunosuppressed or during pregnancy. Group B Streptococci, *Escherichia coli*, and *Klebsiella pneumoniae* can also cause severe meningitis in newborns and infants and are likely introduced orally into the newborn during labor (Pelkonen et al., 2020).

It is notable that if we compare the list of pathogens that commonly cause meningitis in children and adults to those most commonly isolated from blood, there is a disconnect. Aside from neonates, bacteria originating in the gastrointestinal tract are largely well defended by the meninges. For example, in developed countries, patients presenting from the community with bacteremia most frequently present with non-salmonella, gut-derived gram-negative bacilli, such as *E. coli*, *K. pneumoniae*, and *Pseudomonas aeruginosa*, although many of these patients have chronic diseases (diabetes, cancer, organ failure) with associated healthcare exposure (Cross and Levine, 2017; Takeshita et al., 2017). The estimated prevalence of gram-negative bacteremia in the U.K. is 65 per 100,000 for *E. coli* alone (Public Health England, 2021), whereas the prevalence of gram-negative meningitis is <0.5 per 100,000 (McGill et al., 2018). In developing countries, where there is less health care contact and epidemiology may more faithfully reflect evolutionary bacterial pressures, longitudinal studies (e.g., in Malawi) report non-typhoidal salmonella as a leading cause of bacteremia, with *Salmonella typhi* emerging in epidemics (Musicha et al., 2017). However, as in developed countries, *Streptococcus pneumoniae* and *Neisseria meningitidis* are the most common causes of bacterial meningitis (Brown, 1975), aside from the more recent increase in cryptococcal meningitis in the setting of HIV infection (Gordon et al., 2000). We would contend that the fact that gut-derived bacteria are the major source of bacteremia but do not commonly lead to CNS infections is remarkable. Although this discrepancy may, to some extent, reflect intrinsic differences in virulence factors between distinct species of bacteria, e.g., *N. meningitidis*, *S. pneumoniae*, and *H. influenzae* express IgA proteases (Kilian et al., 1979; Mulks et al., 1982; Mulks et al., 1980), it may also be due to linked immunity between gut and meninges. Evolutionarily, enabling the intestine to educate or influence meningeal immunity is an obvious way to prime CNS defense against the most likely blood invaders.

## Gut–meningeal immunity

In considering how meningeal humoral immunity may adapt to the threat of distal mucosal challenge, we recently proposed that the meninges homeostatically recruit intestinal immune cells to defend the walls of the dural venous sinus (Fitzpatrick et al., 2020). Antibody production is at the heart of humoral immune responses, and a function of terminally differentiated B cells termed plasmablasts or plasma cells. The constant region of Igs, comprised exclusively of heavy chain proteins, determines antibody isotype and dictates effector functions. IgA is the predominant antibody isotype produced in intestinal tissue; it exists largely in dimeric form and is often polyreactive such that a single clone can bind multiple microbial species. Luminal IgA can neutralize intestinal pathogens and pathogen-derived toxins, as well as induce enchainment of gut-resident microbes (Bunker et al., 2017; Fagarasan, 2008; Moor et al., 2017).

**Figure 2.**
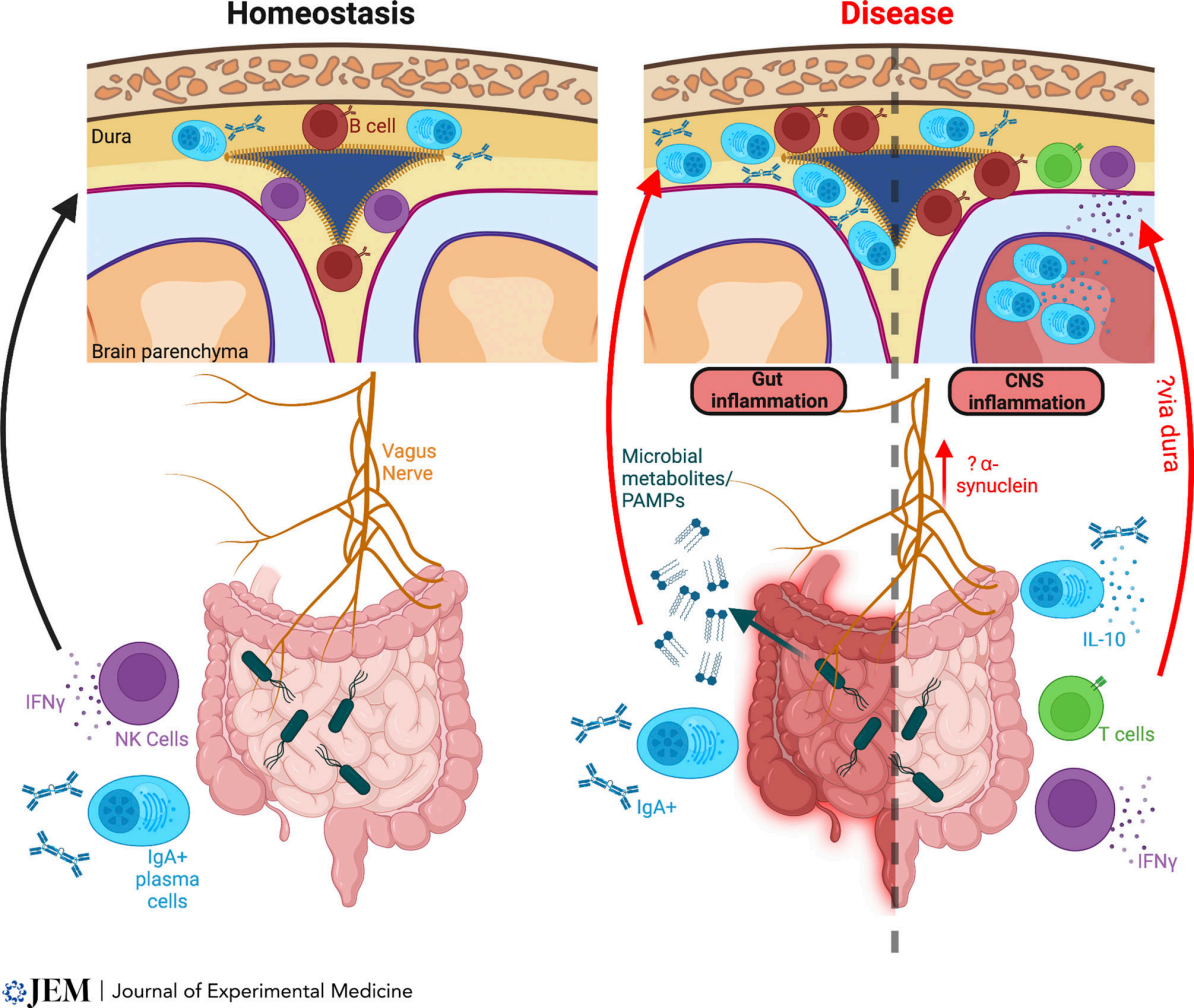
**Gut-meningeal/brain communication.** Homeostasis (left): Gut-educated IgA+ plasma cells localize to the dura, along the walls of the venous sinuses. Their presence is microbiome dependent, being absent in germ-free mice, and restored if the gut microbiome is reconstituted. Intestinal NK cell migration to the meninges is also microbiome dependent, and meningeal NK cells constitutively express IFN-γ in a microbiome-dependent manner. Disease (right): Meningeal IgA plasma cells and B cells increase in number during intestinal inflammation, which may reflect local proliferation as a result of microbial stimuli (PAMPs [pathogen-associated molecular patterns], e.g., LPS) spilling across a leaky gut, or direct migration from the gastrointestinal tract. In CNS inflammation, e.g., EAE or stroke, gut-educated IgA plasma cells migrate to the CNS parenchyma and modulate neuropathology via production of IL-10. Whether they travel through the dura en route to the parenchyma is unknown. In EAE, intestinal NK cells directly migrate from gut to dura, and NK cell–derived IFN-γ leads to an immunoregulatory phenotype in some astrocytes. In a mouse model of stroke, gut-derived T cells migrated to the meninges, whereas B cells migrate to both the meninges and dCLNs. In Parkinson’s disease, microbiome-derived short-chain fatty acids may contribute to microglial activation and motor deficits. Vagus nerve: The vagus nerve arises from the dorsal motor nucleus in the medulla and innervates the gastrointestinal tract, heart, and lungs, and may act as a conduit for gastrointestinal signals to access the CNS, for example gut-derived α-synuclein in Parkinson’s disease. Since it traverses the meninges en route to the CNS, it could potentially carry signals that modulate meningeal immunity.

While assessing the humoral immune landscape in the dura under steady-state conditions, we discovered that, surprisingly, IgA was the dominant Ig isotype expressed in this compartment (Fitzpatrick et al., 2020). A subset of meningeal plasma cells coexpressed joining (J) chain, suggesting that a proportion of these cells may secrete polymeric forms of IgA, akin to intestinal plasma cells. Meningeal IgA^+^ plasma cells were specifically localized to the walls of the dural venous sinuses and were nearly absent in germ-free animals, with their presence restored by gut (but not skin) recolonization. Peri-sinus IgA^+^ plasma cells and B cells increased in number following intestinal barrier breach and B cell receptor sequencing of paired meningeal and intestinal tissue in naive animals, which confirmed a shared repertoire of IgA clones between these geographically distant tissues. Specific deletion of meningeal plasma cells by local application of a proteosome inhibitor led to a reduction in microbial entrapment in the peri-sinus region, enhanced spread into the brain, and increased mortality following intravenous candida challenge. This study revealed a critical role for meningeal IgA in protecting the brain from extrinsic, blood-borne pathogens and demonstrated an important link between humoral immunity in the gut and meninges (Fig. 2). The presence of meningeal IgA plasma cells was further corroborated in a recent study, which also reported an age-associated accumulation of IgG^+^ and IgM^+^ B cells and plasma cells (Brioschi et al., 2021). Thus, the dura is a dynamic barrier tissue that changes its composition in the context of systemic perturbations and normal aging.

Previous reports had described gut-derived IgA plasma cells in the CNS parenchyma in experimental autoimmune encephalitis (EAE), a mouse model of multiple sclerosis, but focused on their antibody-independent functions (Rojas et al., 2019). Plasma cell–deficient mice developed an exacerbated clinical phenotype, whereas mice with elevated numbers of IgA^+^ plasma cells were protected from EAE in an IL-10–dependent manner, suggesting that these cells are important immunoregulators in this context. Moreover, when EAE was induced in mice that had recovered from a previous intestinal rotavirus infection, rotavirus-specific IgA plasma cells were detectable in the CNS parenchyma, although the meninges were not examined in this study (Rojas et al., 2019). An anti-inflammatory role for B cells in EAE has long been noted, with B cell–deficient mice lacking surface IgM expression (µ-MT mice) demonstrating delayed recovery from EAE (Wolf et al., 1996), and B cell depletion may both improve or exacerbate EAE, depending on the timing of administration (Matsushita et al., 2008). We recently showed that meningeal B cells may have anti-inflammatory functions in homeostasis and in the context of psychological stress, with CD19-deficient mice demonstrating meningeal myeloid cell activation in homeostasis and a more anxious phenotype, as well as increased expression of IFN-γ– and IFN-α–response genes in the dura following chronic social defeat, compared with controls (Lynall et al., 2021). Therefore, the intestinal education of humoral immunity may have two beneficial effects for CNS defense: producing cells with B cell receptor affinity matured to recognize the likeliest source of blood-borne bacteria and those with an immunoregulatory cytokine profile that can limit CNS inflammation (Fig. 2).

There is also evidence to suggest that other immune cells, beyond B cells and plasma cells, may be recruited from the gut to the CNS in response to a CNS-intrinsic challenge. In a mouse model of stroke, intestinal photoconversion was used to trace the migration of B and T cells from the gut to the meninges (Benakis et al., 2016). While B cells appeared to home to the meninges and the deep and the superficial CLNs, T cells preferentially homed to the meninges. Similarly, a study of meningeal NK cells and their role in regulating a key subset of astrocytes during EAE, utilized intestinal photoconversion to show evidence of NK cell migration from the gut to the meninges (Sanmarco et al., 2021). NK cell migration was lower in mice receiving adoptively transferred splenocytes from germ-free mice compared with splenocytes from specific pathogen–free (SPF) mice, showing the importance of the microbiome in influencing this phenomenon. Finally, a study of rare brain parenchymal CD4^+^ T cells found that SPF mice cohoused with “dirty” mice had higher numbers of brain parenchymal T cells than SPF mice, suggesting that the gut microbiome either directly or indirectly influences CNS T cell number (Pasciuto et al., 2020). Thus, a number of studies provide direct and indirect evidence of homeostatic, and perturbation-inducible recruitment of intestinal lymphocytes to the CNS and meninges. Such recruitment in vivo may protect the host from the development of infective meningitis in the context of bacteremia.

In addition to supplying the meninges with gut-educated immune cells, there are several other mechanisms by which the intestine may influence meningeal immune cells; blood-borne metabolites and pathogen-associated molecular patterns arising from the intestinal microbiome may reach dural cells via its fenestrated vasculature. In keeping with this concept, transplantation of Parkinson’s disease patient microbiome into α-synuclein transgenic mice led to accelerated disease progression, proposed to be due to the effects of microbial-derived short-chain fatty acids (Sampson et al., 2016). In addition, the vagus nerve directly supplies the gut, traveling through the meninges on its journey. An increasing number of studies suggest that this conduit may support the retrograde transmission of immunological information back into the CNS (Breit et al., 2018; Houser and Tansey, 2017), with the potential to communicate with meningeal immune cells en route (Fig. 2).

## Conclusion

With its substantive populations of IgA-producing plasma cells, as well as ILC2, γδ17, and antigen-experienced T cells, including T regs, the immune composition of the peri-sinusoidal dura evokes notable comparisons with the intestinal lamina propria. The gut is an organ containing trillions of microorganisms, including bacteria, fungi, and viruses, many of which have beneficial effects, and must therefore be tolerated by the intestinal immune system, which is rich in regulatory cells. However, this intimate relationship and close proximity to the microbiome inevitably comes with risks, with intestinal organisms the most common cause of bacteremia. The vasculature of the meninges and CNS contain several areas with fenestrated endothelium, including regions of the dural venous sinuses that also have a low flow rate of blood. These factors confer vulnerability to invasion by circulating microbes. We propose that this has evolutionarily led to the establishment of close links between the gut and meninges, to prime the CNS defense against the most likely invaders. This paradigm is exemplified by the dural venous sinus IgA defense system, where the antibody repertoire mirrors that of the gut. It is notable that the bacterial species that overcome this defense system to cause meningitis both originate from a non-gut source and have evolved to express IgA-cleaving proteases (Kilian et al., 1979; Mulks et al., 1982; Mulks et al., 1980). The immunoregulatory profile of gut immune cells may also have benefits for the CNS in limiting inflammation and the collateral damage associated with it. Although there have been a number of exciting breakthroughs in the area in recent years, a number of questions remain to be resolved: What are the cues guiding intestinal immune cells to the dura? Where do they enter? To what extent do gut-derived immune cells move from the dura to the brain and spinal cord in response to different CNS-intrinsic perturbations, and is this process helpful or harmful? How do microbial metabolites influence different meningeal immune cell subsets? Answers to these questions will no doubt emerge in this dynamic area of immunology, and may well reveal how this axis could be harnessed or attenuated therapeutically in neurological and psychiatric diseases.

## References

[bib1] Adriani, K.S., D. van de Beek, M.C. Brouwer, L. Spanjaard, and J. de Gans. 2007. Community-acquired recurrent bacterial meningitis in adults. Clin. Infect. Dis. 45:e46–e51. 10.1086/520682 17682979

[bib2] Ahn, J.H., H. Cho, J.H. Kim, S.H. Kim, J.S. Ham, I. Park, S.H. Suh, S.P. Hong, J.H. Song, Y.K. Hong, . 2019. Meningeal lymphatic vessels at the skull base drain cerebrospinal fluid. Nature. 572:62–66. 10.1038/s41586-019-1419-5 31341278

[bib3] Alcolado, R., R.O. Weller, E.P. Parrish, and D. Garrod. 1988. The cranial arachnoid and pia mater in man: anatomical and ultrastructural observations. Neuropathol. Appl. Neurobiol. 14:1–17. 10.1111/j.1365-2990.1988.tb00862.x 3374751

[bib4] Alves de Lima, K., J. Rustenhoven, and J. Kipnis. 2020. Meningeal immunity and its function in maintenance of the central nervous system in health and disease. Annu. Rev. Immunol. 38:597–620. 10.1146/annurev-immunol-102319-103410 32340575

[bib5] Antila, S., S. Karaman, H. Nurmi, M. Airavaara, M.H. Voutilainen, T. Mathivet, D. Chilov, Z. Li, T. Koppinen, J.H. Park, . 2017. Development and plasticity of meningeal lymphatic vessels. J. Exp. Med. 214:3645–3667. 10.1084/jem.20170391 29141865 PMC5716035

[bib6] Aspelund, A., S. Antila, S.T. Proulx, T.V. Karlsen, S. Karaman, M. Detmar, H. Wiig, and K. Alitalo. 2015. A dural lymphatic vascular system that drains brain interstitial fluid and macromolecules. J. Exp. Med. 212:991–999. 10.1084/jem.20142290 26077718 PMC4493418

[bib7] Baer, E.T. 2006. Post-dural puncture bacterial meningitis. Anesthesiology. 105:381–393. 10.1097/00000542-200608000-00022 16871073

[bib8] Balin, B.J., R.D. Broadwell, M. Salcman, and M. el-Kalliny. 1986. Avenues for entry of peripherally administered protein to the central nervous system in mouse, rat, and squirrel monkey. J. Comp. Neurol. 251:260–280. 10.1002/cne.902510209 3782501

[bib9] Barry, C., G. Rahmani, and D. Bergin. 2019. Pneumocephalus and meningitis as complications of mastoiditis. Case Rep. Radiol. 2019:7876494. 10.1155/2019/7876494 30915252 PMC6399545

[bib10] Benakis, C., D. Brea, S. Caballero, G. Faraco, J. Moore, M. Murphy, G. Sita, G. Racchumi, L. Ling, E.G. Pamer, . 2016. Commensal microbiota affects ischemic stroke outcome by regulating intestinal γδ T cells. Nat. Med. 22:516–523. 10.1038/nm.4068 27019327 PMC4860105

[bib11] Breit, S., A. Kupferberg, G. Rogler, and G. Hasler. 2018. Vagus nerve as modulator of the brain-gut axis in psychiatric and inflammatory disorders. Front. Psychiatry. 9:44. 10.3389/fpsyt.2018.00044 29593576 PMC5859128

[bib12] Brioschi, S., W.L. Wang, V. Peng, M. Wang, I. Shchukina, Z.J. Greenberg, J.K. Bando, N. Jaeger, R.S. Czepielewski, A. Swain, . 2021. Heterogeneity of meningeal B cells reveals a lymphopoietic niche at the CNS borders. Science. 373:eabf9277. 10.1126/science.abf9277 34083450 PMC8448524

[bib13] Brown, K.G. 1975. Meningitis in Queen Elizabeth Central Hospital, Blantyre, Malawi. East Afr. Med. J. 52:376–385. 1164905

[bib14] Bunker, J.J., S.A. Erickson, T.M. Flynn, C. Henry, J.C. Koval, M. Meisel, B. Jabri, D.A. Antonopoulos, P.C. Wilson, and A. Bendelac. 2017. Natural polyreactive IgA antibodies coat the intestinal microbiota. Science. 358:eaan6619. 10.1126/science.aan6619 28971969 PMC5790183

[bib15] Cai, R., C. Pan, A. Ghasemigharagoz, M.I. Todorov, B. Forstera, S. Zhao, H.S. Bhatia, A. Parra-Damas, L. Mrowka, D. Theodorou, . 2019. Panoptic imaging of transparent mice reveals whole-body neuronal projections and skull-meninges connections. Nat. Neurosci. 22:317–327. 10.1038/s41593-018-0301-3 30598527 PMC6494982

[bib16] Cross, A., and M.M. Levine. 2017. Patterns of bacteraemia aetiology. Lancet Infect. Dis. 17:1005–1006. 10.1016/S1473-3099(17)30491-7 28818542

[bib17] Cugurra, A., T. Mamuladze, J. Rustenhoven, T. Dykstra, G. Beroshvili, Z.J. Greenberg, W. Baker, Z. Papadopoulos, A. Drieu, S. Blackburn, . 2021. Skull and vertebral bone marrow are myeloid cell reservoirs for the meninges and CNS parenchyma. Science. 373:eabf7844. 10.1126/science.abf7844 34083447 PMC8863069

[bib18] Da Mesquita, S., A. Louveau, A. Vaccari, I. Smirnov, R.C. Cornelison, K.M. Kingsmore, C. Contarino, S. Onengut-Gumuscu, E. Farber, D. Raper, . 2018. Functional aspects of meningeal lymphatics in ageing and Alzheimer’s disease. Nature. 560:185–191. 10.1038/s41586-018-0368-8 30046111 PMC6085146

[bib19] Da Mesquita, S., Z. Papadopoulos, T. Dykstra, L. Brase, F.G. Farias, M. Wall, H. Jiang, C.D. Kodira, K.A. de Lima, J. Herz, . 2021. Meningeal lymphatics affect microglia responses and anti-Aβ immunotherapy. Nature. 593:255–260. 10.1038/s41586-021-03489-0 33911285 PMC8817786

[bib20] Dejana, E. 2004. Endothelial cell-cell junctions: happy together. Nat. Rev. Mol. Cell Biol. 5:261–270. 10.1038/nrm1357 15071551

[bib21] Derk, J., H.E. Jones, C. Como, B. Pawlikowski, and J.A. Siegenthaler. 2021. Living on the edge of the CNS: meninges cell diversity in health and disease. Front. Cell. Neurosci. 15:703944. 10.3389/fncel.2021.703944 34276313 PMC8281977

[bib22] Fagarasan, S. 2008. Evolution, development, mechanism and function of IgA in the gut. Curr. Opin. Immunol. 20:170–177. 10.1016/j.coi.2008.04.002 18456485

[bib23] Fitzpatrick, Z., G. Frazer, A. Ferro, S. Clare, N. Bouladoux, J. Ferdinand, Z.K. Tuong, M.L. Negro-Demontel, N. Kumar, O. Suchanek, . 2020. Gut-educated IgA plasma cells defend the meningeal venous sinuses. Nature. 587:472–476. 10.1038/s41586-020-2886-4 33149302 PMC7748383

[bib24] Gadani, S.P., I. Smirnov, A.T. Wiltbank, C.C. Overall, and J. Kipnis. 2017. Characterization of meningeal type 2 innate lymphocytes and their response to CNS injury. J. Exp. Med. 214:285–296. 10.1084/jem.20161982 27994070 PMC5294864

[bib25] GBD 2016 Meningitis Collaborators. 2018. Global, regional, and national burden of meningitis, 1990-2016: a systematic analysis for the Global Burden of Disease Study 2016. Lancet Neurol. 17:1061–1082. 10.1016/S1474-4422(18)30387-9 30507391 PMC6234314

[bib26] Gordon, S.B., A.L. Walsh, M. Chaponda, M.A. Gordon, D. Soko, M. Mbwvinji, M.E. Molyneux, and R.C. Read. 2000. Bacterial meningitis in Malawian adults: pneumococcal disease is common, severe, and seasonal. Clin. Infect. Dis. 31:53–57. 10.1086/313910 10913396

[bib27] Hannocks, M.J., M.E. Pizzo, J. Huppert, T. Deshpande, N.J. Abbott, R.G. Thorne, and L. Sorokin. 2018. Molecular characterization of perivascular drainage pathways in the murine brain. J. Cereb. Blood Flow Metab. 38:669–686. 10.1177/0271678X17749689 29283289 PMC5888861

[bib28] Herisson, F., V. Frodermann, G. Courties, D. Rohde, Y. Sun, K. Vandoorne, G.R. Wojtkiewicz, G.S. Masson, C. Vinegoni, J. Kim, . 2018. Direct vascular channels connect skull bone marrow and the brain surface enabling myeloid cell migration. Nat. Neurosci. 21:1209–1217. 10.1038/s41593-018-0213-2 30150661 PMC6148759

[bib29] Houser, M.C., and M.G. Tansey. 2017. The gut-brain axis: is intestinal inflammation a silent driver of Parkinson’s disease pathogenesis? NPJ Parkinsons Dis. 3:3. 10.1038/s41531-016-0002-0 28649603 PMC5445611

[bib30] Iliff, J.J., M. Wang, Y. Liao, B.A. Plogg, W. Peng, G.A. Gundersen, H. Benveniste, G.E. Vates, R. Deane, S.A. Goldman, . 2012. A paravascular pathway facilitates CSF flow through the brain parenchyma and the clearance of interstitial solutes, including amyloid β. Sci. Transl. Med. 4:147ra111. 10.1126/scitranslmed.3003748 PMC3551275 22896675

[bib31] Kierdorf, K., T. Masuda, M.J.C. Jordao, and M. Prinz. 2019. Macrophages at CNS interfaces: ontogeny and function in health and disease. Nat. Rev. Neurosci. 20:547–562. 10.1038/s41583-019-0201-x 31358892

[bib32] Kilian, M., J. Mestecky, and R.E. Schrohenloher. 1979. Pathogenic species of the genus *Haemophilus* and *Streptococcus pneumoniae* produce immunoglobulin A1 protease. Infect. Immun. 26:143–149. 10.1128/iai.26.1.143-149.1979 40878 PMC414586

[bib33] Kilic, T., and A. Akakin. 2008. Anatomy of cerebral veins and sinuses. Front. Neurol. Neurosci. 23:4–15. 10.1159/000111256 18004050

[bib34] Korin, B., T.L. Ben-Shaanan, M. Schiller, T. Dubovik, H. Azulay-Debby, N.T. Boshnak, T. Koren, and A. Rolls. 2017. High-dimensional, single-cell characterization of the brain’s immune compartment. Nat. Neurosci. 20:1300–1309. 10.1038/nn.4610 28758994

[bib35] Louveau, A., J. Herz, M.N. Alme, A.F. Salvador, M.Q. Dong, K.E. Viar, S.G. Herod, J. Knopp, J.C. Setliff, A.L. Lupi, . 2018. CNS lymphatic drainage and neuroinflammation are regulated by meningeal lymphatic vasculature. Nat. Neurosci. 21:1380–1391. 10.1038/s41593-018-0227-9 30224810 PMC6214619

[bib36] Louveau, A., I. Smirnov, T.J. Keyes, J.D. Eccles, S.J. Rouhani, J.D. Peske, N.C. Derecki, D. Castle, J.W. Mandell, K.S. Lee, . 2015. Structural and functional features of central nervous system lymphatic vessels. Nature. 523:337–341. 10.1038/nature14432 26030524 PMC4506234

[bib37] Lun, M.P., E.S. Monuki, and M.K. Lehtinen. 2015. Development and functions of the choroid plexus-cerebrospinal fluid system. Nat. Rev. Neurosci. 16:445–457. 10.1038/nrn3921 26174708 PMC4629451

[bib38] Lynall, M.E., S.L. Kigar, M.L. Lehmann, A.E. DePuyt, Z.K. Tuong, S.J. Listwak, A.G. Elkahloun, E.T. Bullmore, M. Herkenham, and M.R. Clatworthy. 2021. B-cells are abnormal in psychosocial stress and regulate meningeal myeloid cell activation. Brain Behav. Immun. 97:226–238. 10.1016/j.bbi.2021.08.002 34371135 PMC8453122

[bib39] Ma, Q., B.V. Ineichen, M. Detmar, and S.T. Proulx. 2017. Outflow of cerebrospinal fluid is predominantly through lymphatic vessels and is reduced in aged mice. Nat. Commun. 8:1434. 10.1038/s41467-017-01484-6 29127332 PMC5681558

[bib40] Mastorakos, P., and D. McGavern. 2019. The anatomy and immunology of vasculature in the central nervous system. Sci. Immunol. 4:eaav0492. 10.1126/sciimmunol.aav0492 31300479 PMC6816468

[bib41] Mastorakos, P., M.V. Russo, T. Zhou, K. Johnson, and D.B. McGavern. 2021. Antimicrobial immunity impedes CNS vascular repair following brain injury. Nat. Immunol. 22:1280–1293. 10.1038/s41590-021-01012-1 34556874 PMC8488012

[bib42] Matsushita, T., K. Yanaba, J.D. Bouaziz, M. Fujimoto, and T.F. Tedder. 2008. Regulatory B cells inhibit EAE initiation in mice while other B cells promote disease progression. J. Clin. Invest. 118:3420–3430. 10.1172/JCI36030 18802481 PMC2542851

[bib43] McGill, F., M.J. Griffiths, L.J. Bonnett, A.M. Geretti, B.D. Michael, N.J. Beeching, D. McKee, P. Scarlett, I.J. Hart, K.J. Mutton, . 2018. Incidence, aetiology, and sequelae of viral meningitis in UK adults: a multicentre prospective observational cohort study. Lancet Infect. Dis. 18:992–1003. 10.1016/S1473-3099(18)30245-7 30153934 PMC6105576

[bib44] Moor, K., M. Diard, M.E. Sellin, B. Felmy, S.Y. Wotzka, A. Toska, E. Bakkeren, M. Arnoldini, F. Bansept, A.D. Co, . 2017. High-avidity IgA protects the intestine by enchaining growing bacteria. Nature. 544:498–502. 10.1038/nature22058 28405025

[bib45] Mortazavi, M.M., R.S. Tubbs, S. Riech, K. Verma, M.M. Shoja, A. Zurada, B. Benninger, M. Loukas, and A.A. Cohen Gadol. 2012. Anatomy and pathology of the cranial emissary veins: a review with surgical implications. Neurosurgery. 70:1312–1318; discussion 1318–1319. 10.1227/NEU.0b013e31824388f8 22127046

[bib46] Mrdjen, D., A. Pavlovic, F.J. Hartmann, B. Schreiner, S.G. Utz, B.P. Leung, I. Lelios, F.L. Heppner, J. Kipnis, D. Merkler, . 2018. High-dimensional single-cell mapping of central nervous system immune cells reveals distinct myeloid subsets in health, aging, and disease. Immunity. 48:599. 10.1016/j.immuni.2018.02.014 29562204

[bib47] Mulks, M.H., S.J. Kornfeld, B. Frangione, and A.G. Plaut. 1982. Relationship between the specificity of IgA proteases and serotypes in *Haemophilus influenzae*. J. Infect. Dis. 146:266–274. 10.1093/infdis/146.2.266 6809843

[bib48] Mulks, M.H., A.G. Plaut, H.A. Feldman, and B. Frangione. 1980. IgA proteases of two distinct specificities are released by *Neisseria meningitidis*. J. Exp. Med. 152:1442–1447. 10.1084/jem.152.5.1442 6776228 PMC2185987

[bib49] Musicha, P., J.E. Cornick, N. Bar-Zeev, N. French, C. Masesa, B. Denis, N. Kennedy, J. Mallewa, M.A. Gordon, C.L. Msefula, . 2017. Trends in antimicrobial resistance in bloodstream infection isolates at a large urban hospital in Malawi (1998-2016): a surveillance study. Lancet Infect. Dis. 17:1042–1052. 10.1016/S1473-3099(17)30394-8 28818544 PMC5610140

[bib50] Nabeshima, S., T.S. Reese, D.M. Landis, and M.W. Brightman. 1975. Junctions in the meninges and marginal glia. J. Comp. Neurol. 164:127–169. 10.1002/cne.901640202 810497

[bib51] Pasciuto, E., O.T. Burton, C.P. Roca, V. Lagou, W.D. Rajan, T. Theys, R. Mancuso, R.Y. Tito, L. Kouser, Z. Callaerts-Vegh, . 2020. Microglia require CD4 T cells to complete the fetal-to-adult transition. Cell. 182:625–640.e24. 10.1016/j.cell.2020.06.026 32702313 PMC7427333

[bib52] Pelkonen, T., S. Urtti, E. Dos Anjos, O. Cardoso, L. de Gouveia, I. Roine, H. Peltola, A. von Gottberg, and M.H. Kyaw. 2020. Aetiology of bacterial meningitis in infants aged <90 days: prospective surveillance in Luanda, Angola. Int. J. Infect. Dis. 97:251–257. 10.1016/j.ijid.2020.06.016 32534141

[bib53] Proulx, S.T. 2021. Cerebrospinal fluid outflow: a review of the historical and contemporary evidence for arachnoid villi, perineural routes, and dural lymphatics. Cell Mol. Life Sci. 78:2429–2457. 10.1007/s00018-020-03706-5 33427948 PMC8004496

[bib54] Public Health England. 2021. Annual epidemiological commentary: Gram-negative bacteraemia, MRSA bacteraemia, MSSA bacteraemia and C. difficile infections, up to and including financial year April 2020 to March 2021. https://assets.publishing.service.gov.uk/government/uploads/system/uploads/attachment_data/file/1016843/Annual_epidemiology_commentary_April_2020_March_2021.pdf

[bib55] Ribeiro, M., H.C. Brigas, M. Temido-Ferreira, P.A. Pousinha, T. Regen, C. Santa, J.E. Coelho, I. Marques-Morgado, C.A. Valente, S. Omenetti, . 2019. Meningeal γδ T cell-derived IL-17 controls synaptic plasticity and short-term memory. Sci. Immunol. 4:eaay5199. 10.1126/sciimmunol.aay5199 31604844 PMC6894940

[bib56] Ringstad, G., and P.K. Eide. 2020. Cerebrospinal fluid tracer efflux to parasagittal dura in humans. Nat. Commun. 11:354. 10.1038/s41467-019-14195-x 31953399 PMC6969040

[bib57] Rojas, O.L., A.K. Pröbstel, E.A. Porfilio, A.A. Wang, M. Charabati, T. Sun, D.S.W. Lee, G. Galicia, V. Ramaglia, L.A. Ward, . 2019. Recirculating intestinal IgA-producing cells regulate neuroinflammation via IL-10. Cell. 176:610–624.e18. 10.1016/j.cell.2018.11.035 30612739 PMC6903689

[bib58] Roland, J., C. Bernard, S. Bracard, A. Czorny, J. Floquet, J.M. Race, P. Forlodou, and L. Picard. 1987. Microvascularization of the intracranial dura mater. Surg. Radiol. Anat. 9:43–49. 10.1007/BF02116853 3112977

[bib59] Rua, R., J.Y. Lee, A.B. Silva, I.S. Swafford, D. Maric, K.R. Johnson, and D.B. McGavern. 2019. Infection drives meningeal engraftment by inflammatory monocytes that impairs CNS immunity. Nat. Immunol. 20:407–419. 10.1038/s41590-019-0344-y 30886419 PMC6481670

[bib60] Rustenhoven, J., A. Drieu, T. Mamuladze, K.A. de Lima, T. Dykstra, M. Wall, Z. Papadopoulos, M. Kanamori, A.F. Salvador, W. Baker, . 2021. Functional characterization of the dural sinuses as a neuroimmune interface. Cell. 184:1000–1016.e27. 10.1016/j.cell.2020.12.040 33508229 PMC8487654

[bib61] Sampson, T.R., J.W. Debelius, T. Thron, S. Janssen, G.G. Shastri, Z.E. Ilhan, C. Challis, C.E. Schretter, S. Rocha, V. Gradinaru, . 2016. Gut microbiota regulate motor deficits and neuroinflammation in a model of Parkinson’s disease. Cell. 167:1469–1480.e12. 10.1016/j.cell.2016.11.018 27912057 PMC5718049

[bib62] Sanmarco, L.M., M.A. Wheeler, C. Gútierrez-Vazquez, C.M. Polonio, M. Linnerbauer, F.A. Pinho-Ribeiro, Z. Li, F. Giovannoni, K.V. Batterman, G. Scalisi, . 2021. Gut-licensed IFNγ^+^ NK cells drive LAMP1^+^TRAIL^+^ anti-inflammatory astrocytes. Nature. 590:473–479. 10.1038/s41586-020-03116-4 33408417 PMC8039910

[bib63] Sato, T., H. Konishi, H. Tamada, K. Nishiwaki, and H. Kiyama. 2021. Morphology, localization, and postnatal development of dural macrophages. Cell Tissue Res. 384:49–58. 10.1007/s00441-020-03346-y 33433687

[bib64] Schafflick, D., J. Wolbert, M. Heming, C. Thomas, M. Hartlehnert, A.L. Borsch, A. Ricci, S. Martin-Salamanca, X. Li, I.N. Lu, . 2021. Single-cell profiling of CNS border compartment leukocytes reveals that B cells and their progenitors reside in non-diseased meninges. Nat. Neurosci. 24:1225–1234. 10.1038/s41593-021-00880-y 34253922

[bib65] Schuchardt, F., L. Schroeder, C. Anastasopoulos, M. Markl, J. Bauerle, A. Hennemuth, J. Drexl, J.M. Valdueza, I. Mader, and A. Harloff. 2015. In vivo analysis of physiological 3D blood flow of cerebral veins. Eur. Radiol. 25:2371–2380. 10.1007/s00330-014-3587-x 25638218

[bib66] Takeshita, N., I. Kawamura, H. Kurai, H. Araoka, A. Yoneyama, T. Fujita, Y. Ainoda, R. Hase, N. Hosokawa, H. Shimanuki, . 2017. Unique characteristics of community-onset healthcare-associated bloodstream infections: a multi-centre prospective surveillance study of bloodstream infections in Japan. J. Hosp. Infect. 96:29–34. 10.1016/j.jhin.2017.02.022 28377180

[bib67] Ter Horst, L., M.C. Brouwer, A. van der Ende, and D. van de Beek. 2020. Community-acquired bacterial meningitis in adults with cerebrospinal fluid leakage. Clin. Infect. Dis. 70:2256–2261. 10.1093/cid/ciz649 31300817 PMC7245152

[bib68] Tsutsumi, S., M. Nakamura, T. Tabuchi, Y. Yasumoto, and M. Ito. 2013. Calvarial diploic venous channels: an anatomic study using high-resolution magnetic resonance imaging. Surg. Radiol. Anat. 35:935–941. 10.1007/s00276-013-1123-3 23625040

[bib69] Unhanand, M., M.M. Mustafa, G.H. McCracken Jr., and J.D. Nelson. 1993. Gram-negative enteric bacillary meningitis: a twenty-one-year experience. J. Pediatr. 122:15–21. 10.1016/s0022-3476(05)83480-8 8419603

[bib70] van de Beek, D., M.C. Brouwer, U. Koedel, and E.C. Wall. 2021. Community-acquired bacterial meningitis. Lancet. 398:1171–1183. 10.1016/S0140-6736(21)00883-7 34303412

[bib71] Van Hove, H., L. Martens, I. Scheyltjens, K. De Vlaminck, A.R. Pombo Antunes, S. De Prijck, N. Vandamme, S. De Schepper, G. Van Isterdael, C.L. Scott, . 2019. A single-cell atlas of mouse brain macrophages reveals unique transcriptional identities shaped by ontogeny and tissue environment. Nat. Neurosci. 22:1021–1035. 10.1038/s41593-019-0393-4 31061494

[bib72] Wang, Y., D. Chen, D. Xu, C. Huang, R. Xing, D. He, and H. Xu. 2021. Early developing B cells undergo negative selection by central nervous system-specific antigens in the meninges. Immunity. 54:2784–2794.e6. 10.1016/j.immuni.2021.09.016 34626548

[bib73] Wolf, S.D., B.N. Dittel, F. Hardardottir, and C.A. Janeway Jr. 1996. Experimental autoimmune encephalomyelitis induction in genetically B cell-deficient mice. J. Exp. Med. 184:2271–2278. 10.1084/jem.184.6.2271 8976182 PMC2196394

[bib74] Yao, H., T.T. Price, G. Cantelli, B. Ngo, M.J. Warner, L. Olivere, S.M. Ridge, E.M. Jablonski, J. Therrien, S. Tannheimer, . 2018. Leukaemia hijacks a neural mechanism to invade the central nervous system. Nature. 560:55–60. 10.1038/s41586-018-0342-5 30022166 PMC10257142

[bib75] Ziegler, A., M. Patadia, and J. Stankiewicz. 2018. Neurological complications of acute and chronic sinusitis. Curr. Neurol. Neurosci. Rep. 18:5. 10.1007/s11910-018-0816-8 29404826

